# Proteomic Profiling of Venoms from *Bungarus suzhenae* and *B. bungaroides*: Enzymatic Activities and Toxicity Assessment

**DOI:** 10.3390/toxins16110494

**Published:** 2024-11-16

**Authors:** Chenying Yang, Li Ding, Qiyi He, Xiya Chen, Haiting Zhu, Feng Chen, Wanzhou Yang, Yuexin Pan, Zhiyuan Tai, Wenhao Zhang, Zeyuan Yu, Zening Chen, Xiaodong Yu

**Affiliations:** 1 Animal Toxin Group, Engineering Research Center of Active Substance and Biotechnology, Ministry of Education, College of Life Science, Chongqing Normal University, Chongqing 401331, China; yangyoungv@163.com (C.Y.); hqy7171@126.com (Q.H.); chenxyamazingyou@163.com (X.C.); 2022110513069@stu.cqnu.edu.cn (H.Z.); 2022210513049@stu.cqnu.edu.cn (F.C.); yangwazhou6@163.com (W.Y.); moonnewpyx@163.com (Y.P.); tzybxzp@163.com (Z.T.); 2023110513068@stu.cqnu.edu.cn (W.Z.); yuzeyuan123@foxmail.com (Z.Y.); 2 Laboratory of Amphibians and Reptiles, Chengdu Institute of Biology, Chinese Academy of Sciences, Chengdu 610213, China; 3 Key Laboratory of Ecology of Rare and Endangered Species and Environmental Protection, Guangxi Normal University, Ministry of Education, Guilin 541006, China; chenzn@gxnu.edu.cn

**Keywords:** *B. suzhenae*, *B. bungaroides*, proteomics, snake venom

## Abstract

Kraits are venomous snakes of the genus *Bungarus* from the family *Elapidae*. Their venom typically demonstrates neurotoxicity; however, the toxicity is significantly influenced by the snake’s species and geographical origin. Among the *Bungarus* species, *Bungarus suzhenae* and *B. bungaroides* have been poorly studied, with little to no information available regarding their venom composition. In this study, a proteomic approach was employed using LC-MS/MS to identify proteins from trypsin-digested peptides. The analysis revealed 102 venom-related proteins from 18 distinct functional protein families in the venom of *B. suzhenae*, with the primary components being three-finger toxins (3-FTx, 25.84%), phospholipase A_2_ (PLA_2_, 40.29%), L-amino acid oxidase (LAAO, 10.33%), Kunitz-type serine protease inhibitors (KUN, 9.48%), and snake venom metalloproteinases (SVMPs, 6.13%). In the venom of *B. bungaroides*, 99 proteins from 17 families were identified, with primary components being 3-FTx (33.87%), PLA_2_ (37.91%), LAAO (4.21%), and KUN (16.60%). Enzymatic activity assays confirmed the presence of key venom enzymes. Additionally, the LD50 values for *B. suzhenae* and *B. bungaroides* were 0.0133 μg/g and 0.752 μg/g, respectively, providing a reference for toxicity studies of these two species. This research elucidates the proteomic differences in the venoms of these two species, offering a foundation for developing antivenoms and clinical treatments for envenomation.

## 1. Introduction

Venomous snakes secrete a sophisticated and highly potent blend of toxins in the form of venom for purposes of predation, digestion and defense, which is indispensable for their survival. Annually, around 5.4 million snakebite incidents are documented worldwide, resulting in 81,000 to 138,000 deaths due to associated complications, creating significant socioeconomic impacts [1,2,3]. In 2017, the World Health Organization (WHO) recognized snakebites as a neglected tropical disease in urgent need of a global response [4].

The kraits (genus: *Bungarus*), which belong to the family *Elapidae*, are distributed throughout tropical and subtropical Asia. Their range extends from near Iran through the Indian subcontinent, Sri Lanka, Southeast Asia, and Borneo, all the way to southern China and eastern Taiwan [5]. Until now, sixteen species have been identified within this genus. The predominant toxic feature of krait venom is its neurotoxicity, which is attributed to two main types of neurotoxins: presynaptic and postsynaptic toxins [6,7]. Presynaptic neurotoxins disrupt the release of the neurotransmitter acetylcholine from nerve endings [8], while postsynaptic neurotoxins inhibit the binding of acetylcholine to nicotinic receptors on skeletal muscles [9]. Notably, *B. caeruleus* in South Asia, *B. candidus* in Southeast Asia, and *B. multicinctus* in East Asia have been classified as medically significant snakes by the WHO due to the severe snakebite incidents they cause in their respective regions [10,11]. While the venom characteristics of these kraits have been extensively studied, many lesser-known species remain poorly understood.

**Figure 1:**
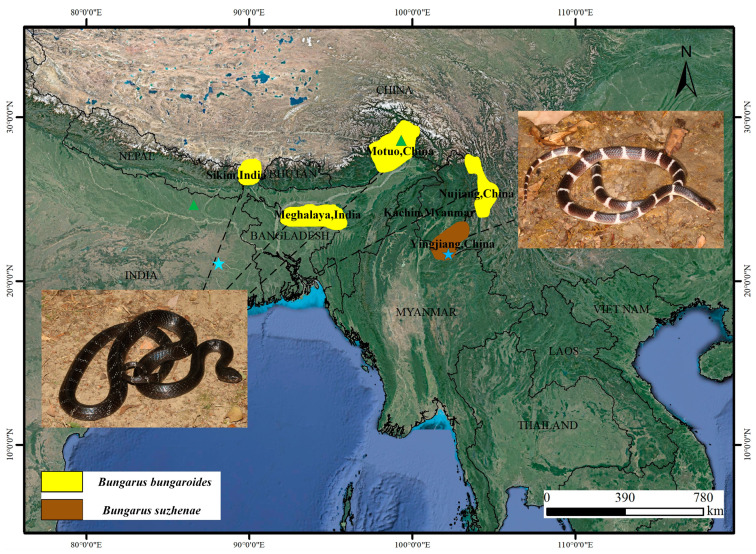
Distribution ranges of *B. suzhenae* and *B. bungaroides*. The brown area represents the distribution of *B. suzhenae*, which is primarily found in Yingjiang County, Dehong Prefecture, Yunnan Province, China, and Kachin State, Myanmar. The yellow area indicates the distribution of *B. bungaroides*, which is mainly located in Sikkim and Meghalaya in India, Nujiang Lisu Autonomous Prefecture, Yunnan Province, and Medog County in Tibet, China. Blue star and green triangle on the map denote sampling points for *B. suzhenae* and *B. bungaroides*, respectively.

*B. suzhenae*, a recently described species in 2021, which killed a famous American Herpetologist Joseph Slowinski in 2001 carrying out fieldwork [12], is primarily distributed in Yingjiang County, Dehong Prefecture, Yunnan Province, China and Kachin State, Myanmar, inhabiting rice paddies and streams in monsoon forests at altitudes between 700 and 1560 m (Figure 1). This species preys on eels (*Monopterus albus*) and small snakes such as *Xenochrophis flavipunctatus* and *Pareas*. Morphologically similar to *B. multicinctus*, *B. suzhenae* is distinguished by having more subcaudal scales and brown or black flecks within its white dorsal bands. Notably, envenomation by *B. suzhenae* differs from *B. multicinctus*, causing not only neuroparalysis symptoms like respiratory distress and blurred speech but also severe pain and necrosis at the bite site.

Additionally, *B. bungaroides* is a rare species, distributed across the Indian states of Sikkim and Meghalaya, as well as in Nujiang Lisu Autonomous Prefecture, Yunnan Province and Medog County, Tibet, China, as shown in Figure 1. It inhabits subtropical forests below 2000 m and, like other kraits, is nocturnal and primarily feeds on other snakes [13]. Morphologically distinct, *B. bungaroides* has narrow white or pale yellow rings on its dark brown to black body, making it one of the most visually unique kraits [14]. To date, no studies have been conducted on the venom toxicity of this species, and no envenomation cases have been reported.

Over the past decade, advancements in “omics” technologies, particularly high-throughput transcriptomics and proteomics of snake venom gland tissues, have dramatically improved our understanding of venom composition, immunogenicity, and toxicity [15,16,17,18,19,20]. These sensitive techniques now allow for in-depth analysis of snake venom gland transcriptomes or proteomes. For instance, recent proteomic studies by Ariadna identified the venom components of three endemic Colombian coral snakes (*Micrurus helleri*, *Micrurus medemi* and *Micrurus sangilensis*) [21], while Tomohisa et al. employed affinity chromatography-based proteomics to analyze the venom of *Protobothrops flavoviridis*, revealing a novel muscle necrosis-enhancing serine protease [22]. Similarly, Taufikul et al. uncovered common and unique pathways involved in Russell’s viper venom-induced neurogenesis using transcriptomic and functional proteomics [23].

In this study, we focused on *B. suzhenae* and *B. bungaroides*, employing bottom-up proteomics and LC-MS/MS to perform a comparative analysis of venom composition and relative abundance. We also evaluated the in vitro bioactivity of venom components to validate the findings and proposed potential explanations for envenomation symptoms. The proteomic features and toxicological insights provide a basis for developing treatment strategies for envenomations and improving clinical management of snakebite cases.

## 2. Results

### 2.1. Proteomic Profiling of B. suzhenae and B. bungaroides Venoms

To explore and compare the proteomic features of *B. suzhenae* and *B. bungaroides* venoms, LC-MS/MS was employed, yielding 1684 peptide sequences. These peptides were matched against the *Elapidae* protein database in UniProt, identifying a total of 485 proteins. After refining the data, 121 venom-related proteins were identified, including 56 with annotated sequences and 65 with unannotated sequences. The original peptide sequence information of *B. suzhenae* and *B. bungaroides* is presented in Table S1.

**Figure 2:**
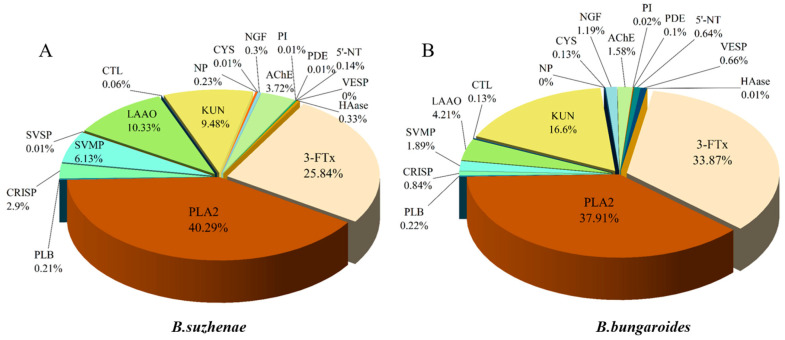
Relative abundance of toxin protein families in the venoms of *B. suzhenae* and *B. bungaroides* as determined by LC-MS/MS. (**A**) Shows the relative abundance of various protein families in *B. suzhenae* venom; (**B**) shows the same for *B. bungaroides* venom. Abbreviations include 3-FTx (three-finger toxin), PLA_2_ (phospholipase A_2_), PLB (phospholipase B), CRISP (cysteine-rich secretory protein), SVMP (snake venom metalloproteinase), SVSP (snake venom serine protease), LAAO (L-amino acid oxidase), CTL (C-type lectin), KUN (Kunitz-type serine protease inhibitor), NP (natriuretic peptide), CYS (cystatin), NGF (nerve growth factor), AChE (acetylcholinesterase), PI (PLA_2_ inhibitor), PDE (phosphodiesterase), 5′-NT (5′-nucleotidase), VESP (vespryn), and HAase (hyaluronidase).

**Figure 3:**
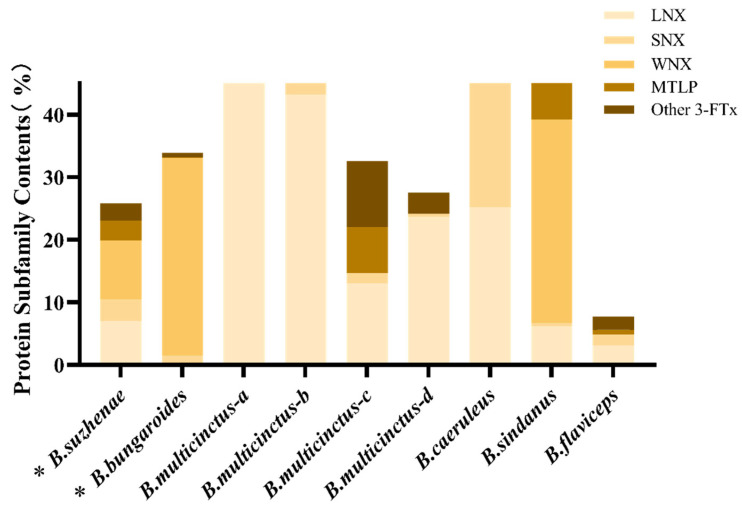
Comparison of the content of 3-FTx protein subfamilies in the venoms of nine Bungarus species. * the ones studied in this research: *B. suzhenae* and *B. bungaroides*. Other species include *B. multicinctus-a* (Guangdong, China) [24], *B. multicinctus-b* (Taiwan, China) [24], *B. multicinctus-c* (Guangxi, China) [25], *B. multicinctus-d* (Vietnam) [26], *B. caeruleus* (India) [27], *B. sindanus* (Pakistan) [28], and *B. flaviceps* (Malaysia) [29]. Subfamilies include LNX (long neurotoxin), SNX (short neurotoxin), WNX (weak neurotoxin), and MTLP (Muscarinic toxin-like proteins).

**Figure 4:**
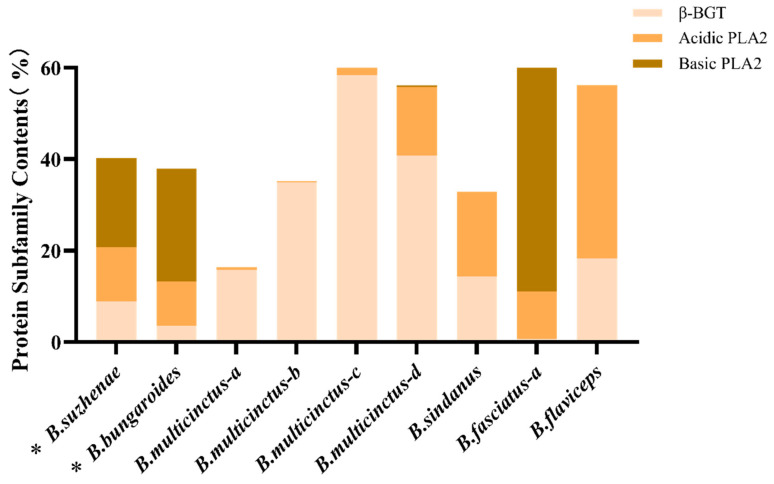
Comparison of the content of PLA_2_ protein subfamilies in the venoms of nine Bungarus species. * the ones studied in this research: *B. suzhenae* and *B. bungaroides*. Other species include *B. multicinctus-a* (Guangdong, China) [24], *B. multicinctus-b* (Taiwan, China) [24], *B. multicinctus-c* (Guangxi, China) [25], *B. multicinctus-d* (Vietnam) [26], *B. sindanus* (Pakistan) [28], *B. fasciatus-a* (Vietnam) [26], and *B. flaviceps* (Malaysia) [29]. Subfamilies include β-BGT (beta-bungarotoxin), Acidic PLA_2_ (acidic phospholipase A_2_), and Basic PLA_2_ (basic phospholipase A_2_).

**Figure 5:**
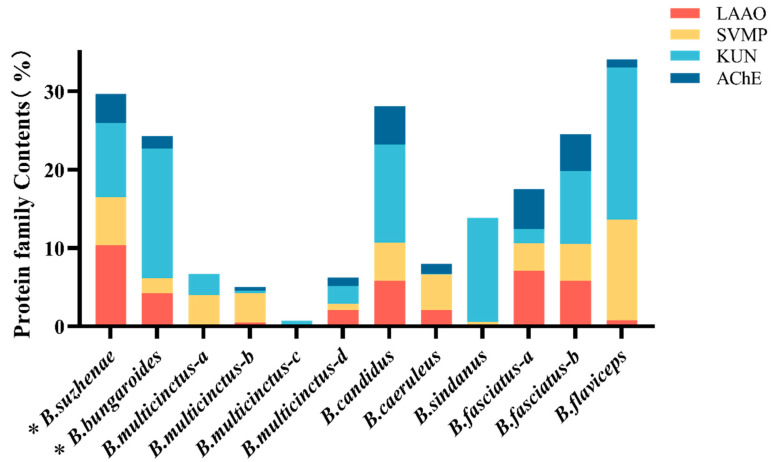
Comparison of minor toxin-related protein families in the venoms of various Bungarus species. Abbreviations include Kunitz-type serine protease inhibitors (KUNs), L-amino acid oxidase (LAAO), snake venom metalloproteinases (SVMPs) and acetylcholinesterase (AChE). * the ones studied in this research: *B. suzhenae* and *B. bungaroides*. Other species include *B. multicinctus-a* (Guangdong, China) [24], *B. multicinctus-b* (Taiwan, China) [24], *B. multicinctus-c* (Guangxi, China) [25], *B. multicinctus-d* (Vietnam) [26], *B. candidus* (Malaysia) [9], *B. caeruleus* (India) [27], *B. sindanus* (Pakistan) [28], *B. fasciatus-a* (Vietnam) [26], *B. fasciatus-b* (Malaysia) [9], and *B. flaviceps* (Malaysia) [29].

In the venom of *B. suzhenae*, proteins were classified into families based on their qualifications, resulting in the identification of 102 proteins from 18 protein families. The most abundant components were three-finger toxins (3-FTx) and phospholipase A_2_ (PLA_2_) (Figure 2, Table 1). As shown in Figure 3, 3-FTx accounts for 25.84% of the total protein content. This group includes long neurotoxins (LNX, 7.02%), short neurotoxins (SNX, 3.48%), muscarinic toxin-like proteins (MTLP, 3.2%), and weak neurotoxins (WNX, 9.39%). PLA_2_ constitutes 40.29% of the total protein and is categorized into three subtypes: β-bungarotoxin (β-BGT, 8.86%), acidic PLA_2_ (11.89%), and basic PLA_2_ (19.54%) (Figure 4). Minor protein families present in both venoms include L-amino acid oxidase (LAAO, 10.33%), snake venom metalloproteinases (SVMPs, 6.13%), Kunitz-type serine protease inhibitors (KUN, 9.48%), and acetylcholinesterase (AChE, 3.72%) (Figure 5). Other protein families, which collectively account for around 4% of the total venom protein content, include cysteine-rich secretory proteins (CRISPs, 2.90%), phospholipase B (PLB, 0.21%), C-type lectins (CTLs, 0.06%), natriuretic peptides (NPs, 0.23%), cysteine protease inhibitors (CYS, 0.01%), nerve growth factors (NGFs, 0.3%), PLA_2_ inhibitors (PIs, 0.01%), phosphodiesterases (PDEs, 0.01%), 5′-nucleotidase (5′-NT, 0.14%), vespryn (VESP, <0.01%), hyaluronidase (HAase, 0.33%), and snake venom serine proteases (SVSPs, 0.01%).

Among the identified protein families, *B. bungaroides* venom contained 99 proteins from 17 families, with three-finger toxins (3-FTx) and phospholipase A_2_ (PLA_2_) also constituting the most abundant components (Figure 2, Table 1). As shown in Figure 3, 3-FTx accounts for 33.87% of the total protein content. This group includes long neurotoxins (LNX, <0.01%), short neurotoxins (SNX, 1.49%), muscarinic toxin-like proteins (MTLPs, 0.15%), and weak neurotoxins (WNX, 31.55%). PLA_2_ makes up 37.91% of the total protein and is categorized into three subtypes: β-bungarotoxin (β-BGT, 3.59%), acidic PLA_2_ (9.62%), and basic PLA_2_ (24.71%) (Figure 4). Minor protein families present in both venoms include LAAO (4.21%), SVMP (1.89%), KUN (16.6%), and AChE (1.58%) (Figure 5). Other protein families, which collectively account for around 4% of the total venom protein content, include CRISP (0.84%), PLB (0.22%), CTL (0.13%), NP (<0.01%), CYS (0.13%), NGF (1.19%), PI (0.02%), PDE (0.1%), 5′-NT (0.64%), VESP (0.66%), and HAase (0.01%). Notably, no snake venom serine proteases (SVSPs) were detected in *B. bungaroides* venom.

### 2.2. Biochemical Characteristics of B. suzhenae and B. bungaroides Venoms

#### 2.2.1. PLA_2_ Activity

**Figure 6:**
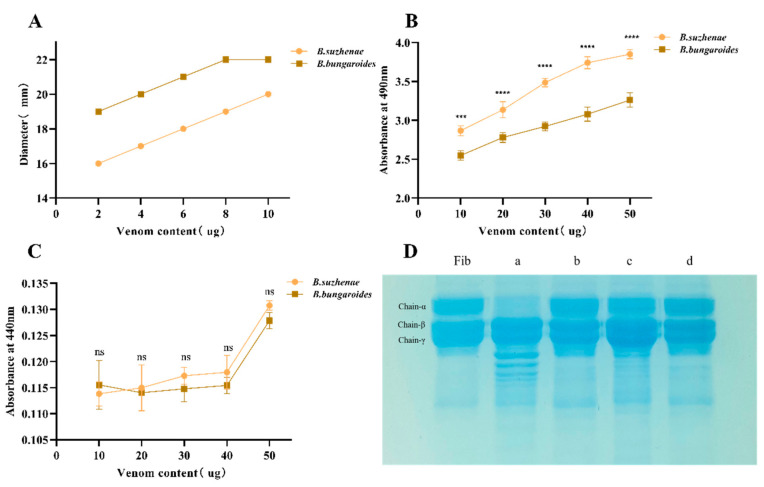
Biochemical activities of venoms from *B. suzhenae* and *B. bungaroides*. This figure depicts the (**A**) PLA_2_, (**B**) LAAO, (**C**) SVMP, and (**D**) Fibrinogen-degrading activities of the venoms. Panel (**D**) shows the fibrinogen electrophoresis patterns: Lane Fib represents untreated fibrinogen, Lane a and b show fibrinogen hydrolysis by *B. suzhenae* venom and by *B. suzhenae* venom with EDTA as an inhibitor, respectively, while Lane c and d show the same for *B. bungaroides* venom. All experiments were performed in triplicate, with error bars representing standard deviation. Statistical significance was assessed with multiple comparison tests: *p* > 0.05 (ns), *p* < 0.0002 (***), *p* < 0.0001 (****).

PLA_2_ was evaluated using lecithin from egg yolk as the substrate. PLA_2_ catalyzes its hydrolysis, producing a clear zone in agar plates, with the diameter of the zone representing the enzyme’s activity in millimeters per diameter (MPD). As shown in Figure 6A, the diameter of the clear zone increased proportionally with the amount of venom. The PLA_2_ activity in *B. bungaroides* venom exhibited an MPD of 4 µg, while *B. suzhenae* venom showed an MPD of 10 µg. These results indicate a distinct difference in the PLA_2_ catalytic activity between the venoms of *B. suzhenae* and *B. bungaroides*. The original gels for the PLA_2_ activity determination of *B. suzhenae* and *B. bungaroides* are depicted in Figure S1.

#### 2.2.2. LAAO Activity

LAAO specifically targets L-amino acids. In this assay, L-leucine was used as the substrate, which was hydrolyzed by LAAO to produce keto acids and H_2_O_2_. The yellow compound generated from the reaction of H_2_O_2_ with OPD (o-phenylenediamine) via HRP oxidation was measured, and the reaction was terminated with sulfuric acid. As depicted in Figure 6B, increasing the venom concentration led to a rise in absorbance values. A significant difference in catalytic activity (*p* < 0.05) was observed between the two venoms at equivalent concentrations, with *B. suzhenae* venom displaying higher LAAO activity compared to *B. bungaroides* venom.

#### 2.2.3. SVMP Hydrolytic Activity

To investigate the proteolytic properties of the venoms from *B. suzhenae* and *B. bungaroides*, azocasein was used as a substrate. Proteins hydrolyzed by snake venom metalloproteinases (SVMPs) generate yellow compounds, which were quantified through a colorimetric assay. As shown in Figure 6C, *B. suzhenae* and *B. bungaroides* venoms exhibited minimal or negligible proteolytic activity.

#### 2.2.4. Fibrinogenolytic Activity

It is well known that snake venom components can affect hemostasis by acting on various factors involved in the blood coagulation cascade. Fibrinogen, a glycoprotein synthesized by hepatocytes, plays a key role in coagulation by promoting platelet aggregation. In SDS-PAGE, fibrinogen presents three distinct bands corresponding to the alpha (Chain-α), beta (Chain-β), and gamma (Chain-γ) chains, arranged from largest to smallest molecular weight. As shown in Figure 6D, after incubating the venoms with fibrinogen at a ratio of 1:10, Lane a illustrates that *B. suzhenae* venom exhibited stronger fibrinogenolytic activity, particularly hydrolyzing Chain-α, whereas Lane c shows weaker fibrinogenolytic activity in *B. bungaroides* venom. Lanes b and d demonstrate the inhibitory effect of EDTA on fibrinogen hydrolysis by both venoms, highlighting the differences in fibrinogenolytic activities between the two species.

#### 2.2.5. Hemolytic Activity

**Figure 7:**
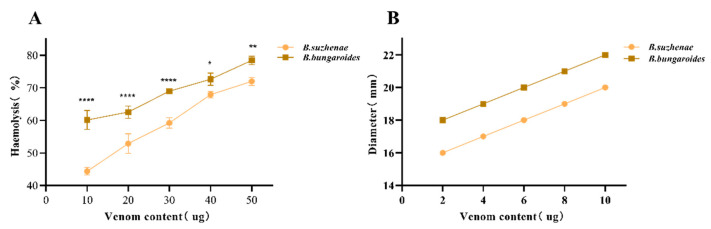
Hemolytic activities of venoms from *B. suzhenae* and *B. bungaroides*. (**A**) Illustrates the direct hemolytic activity of PLA2 on red blood cells, expressed as a percentage in relation to the positive control (1% Triton X). (**B**) Shows the hemolytic effect of phospholipids generated by PLA_2_ hydrolysis on red blood cells. All tests were carried out in triplicate, with error bars denoting standard deviation. Statistical significance was assessed with multiple comparison tests: *p* < 0.05 (*), *p* < 0.002 (**), *p* < 0.0001 (****).

PLA_2_ can induce hemolysis by hydrolyzing phospholipids in cell membranes, disrupting their structure and function, leading to red blood cell lysis; such toxins are considered direct hemolysins. As shown in Figure 7A, increasing venom concentrations led to higher hemolysis rates. Comparatively, *B. bungaroides* venom demonstrated a greater hemolytic activity, with a higher hemolysis rate at the same venom concentration.

PLA_2_-generated lysophospholipids exhibit strong surface activity, which can insert into red blood cell membranes, altering permeability and stability, thereby promoting hemolysis. Additionally, yolk lecithin was utilized as a substrate to represent the hemolysis induced by snake venom through measuring the diameter of the transparent circle and expressed as an MPD value. As shown in Figure 7B, the clear zone increased with venom concentration. *B. bungaroides* venom showed an MPD value of 6 µg, while *B. suzhenae* venom exhibited a higher MPD value of 10 µg. The original gel for the determination of the hemolytic activity of *B. suzhenae* and *B. bungaroides* is presented in Figure S2.

### 2.3. LD_50_

The intravenous LD_50_ values of *B. suzhenae* and *B. bungaroides* venoms were ascertained to be 0.0133 µg/g and 0.752 µg/g, respectively. As shown in Table 2, compared to other identified Bungarus species, *B. suzhenae* venom exhibited the highest lethality, followed by *B. multicinctus*, *B. sindanus*, and *B. candidus*, with *B. bungaroides* venom showing the lowest lethality in this study.

## 3. Discussion

*B. suzhenae* and *B. bungaroides* are two relatively obscure species of kraits, and understanding the precise composition of their venom is of crucial importance for the development of broad-spectrum antivenoms and effective clinical management of snakebite cases. In this study, PLA_2_ was identified as the most abundant component in *B. suzhenae* venom (40.29%), followed by 3-FTx (25.84%) and LAAO (10.33%). In *B. bungaroides* venom, the predominant components were PLA_2_ (37.91%), 3-FTx (33.87%), and KUN (16.6%). As the major constituents of both species’ venom, 3-FTx and PLA_2_ will serve as primary targets for future antivenom development. A comparison with other species within the same genus revealed that while 3-FTx and PLA_2_ are also dominant in those species, the composition and abundance of these protein families differ significantly.

The 3-FTx toxins identified in *B. suzhenae* and *B. bungaroides* venoms primarily belong to subfamilies including long-chain neurotoxins (LNX), short-chain neurotoxins (SNX), weak neurotoxins (WNX), Muscarinic toxin-like proteins (MTLP), and other unconventional orphan subfamilies. Notably, only κ-BGT (O12962) was detected within the LNX family, distinguishing these venoms from other species such as *B. multicinctus*, *B. candidus*, and *B. fasciatus*, where Alpha-bungarotoxin (α-BGT) dominates. κ-BGT primarily targets nicotinic acetylcholine receptors (nAChRs) at the neuromuscular junction, binding to the CHRNA3 subunit and leading to post-synaptic blockade of cholinergic neurotransmission, which weakens muscle contraction and can result in paralysis [33,34,35,36]. Paralysis of respiratory muscles can cause breathing difficulties and even respiratory failure [37]. Previous studies on other krait species reported κ-BGT (Figure 3) in *B. multicinctus* venom from Guangxi, China [25] and Vietnam [26] (2.40–2.47%), with a recent study from Guangdong, China, reporting a higher level of κ-BGT (10.66%) [24].

The MTLP identified in both species’ venoms was Muscarinic toxin BM14 (Q8JFX7), which functions by inhibiting the binding of [^3^H]quinuclidinyl benzilate to M2-type muscarinic acetylcholine receptors (mAChR) [38,39,40]. Although the lethal activity of MTLP is not well studied, it may cause autonomic nervous system dysfunction, manifesting in early symptoms such as abdominal pain or cramping [41,42]. As shown in Figure 3, the content of MTLP in *B. suzhenae* venom (3.20%) was comparable to levels in *B. multicinctus* venom from Taiwan and Guangdong (2.61–2.78%) [24]. In contrast, the content reached 7.07% in *B. sindanus* venom from Pakistan [28]. However, no MTLP has been reported in the venom of *B. caeruleus* [27] or *B. fasciatus* [26]. The SNX family in both venoms comprised proteins such as short neurotoxin homologs (P43445), neurotoxin-like protein (Q7ZT13), short neurotoxin homolog NTL4 (Q9YGI8), and cytotoxin homolog 3 (P01473). These minor 3-FTx-related components have only been characterized based on their amino acid sequences in databases, and their biological activity remains uncharacterized [24].

Research suggests that WNX interacts with both nicotinic and muscarinic acetylcholine receptors [43,44]. The peripheral neuropathic effects of WNX, as demonstrated in mice, are attributed to its action on the visceral nervous system at the plexus-ganglion and postganglionic levels [45], with a much lower lethal dose (LD_50_: 5–80 μg/g) than other neurotoxins [28]. In *B. bungaroides* venom, WNX was identified in significant quantities (31.55%), comprising 93.15% of the total 3-FTx content (Figure 3). A similar proportion of WNX (32.59%) was reported in *B. sindanus* venom from Pakistan [28], though WNX has been less frequently reported in other proteomic studies of krait venoms.

PLA_2_, another major toxin in both *B. suzhenae* and *B. bungaroides* venoms, exhibits catalytic activity by hydrolyzing the ester bond at the sn-2 position of glycerophospholipids, producing free fatty acids and lysophospholipids that disrupt cell membrane integrity [46]. In addition to its enzymatic function, PLA_2_ exhibits a broad range of biological activities, including neurotoxicity, hemotoxicity, myotoxicity, cardiotoxicity, and modulation of platelet aggregation [47,48,49]. As shown in Figure 4, basic PLA_2_ (19.54–24.71%) was significantly more abundant than acidic PLA_2_ (9.62–11.89%) in *B. suzhenae* and *B. bungaroides* venoms. The relative abundance of PLA_2_ varies significantly among species; for instance, a study on *B. flaviceps* venom from Malaysia reported similar PLA_2_ levels (37.7%) [29] to those in our study, whereas *B. fasciatus* venom from Vietnam contained 66.87% [26]. In contrast, non-neurotoxic PLA_2_ levels were much lower in *B. multicinctus* venom from Guangdong, Taiwan [24] and Guangxi, China [25] (0.67%, 0.31%, and 8.05%, respectively).

β-BGT, the most abundant neurotoxin in PLA_2_, is a heterodimer composed of two polypeptide chains with distinct activities [50]. Chain A consists of approximately 120 amino acids and shares structural similarity with PLA_2_, while Chain B is a 61-amino-acid polypeptide resembling the Kunitz-type serine protease inhibitor (KUN) domain [51,52]. β-BGT exerts its presynaptic effects by interfering with Ca^2+^ channels, inhibiting neurotransmitter release and consequently blocking neuromuscular transmission. Severe cases of β-BGT poisoning can result in respiratory muscle paralysis and death [53]. β-BGT has an exceptionally high lethality, with an intravenous LD_50_ of 0.007 μg/g [54]. In this study, β-BGT was more abundant in *B. suzhenae* venom (8.86%) compared to *B. bungaroides* (3.59%) (Figure 4). In *B. multicinctus* venom from Guangxi, China [25] and Vietnam [26], β-BGT constituted approximately 50% of the total venom protein, whereas studies on *B. caeruleus* (12.90%) [27] and *B. flaviceps* (18.27%) [29] reported β-BGT levels comparable to those in this study. However, *B. fasciatus* venom from Vietnam exhibited extremely low levels of β-BGT (0.66%) [26].

KUN, a small protein (50–60 amino acids) containing the Kunitz domain [55], is categorized into neurotoxic and non-neurotoxic types [56]. The neurotoxic group can block ion channels (e.g., potassium and calcium channels) but loses its enzymatic inhibitory function [55]. In this study, KUN proteins in both venoms included neurotoxic forms, comprising 7.74% of *B. suzhenae* venom and 16.13% of *B. bungaroides* venom (Figure 5). Compared with other species within the same genus, KUN levels were also high in *B. candidus* (12.50%) [9], *B. sindanus* (13.30%) [28], and *B. flaviceps* (19.40%) [29], but relatively lower in *B. caeruleus* and *B. multicinctus* (<3%) [24,25,26,27]. The presence of both the A subunit and the B subunit of β-BGT in the venoms of *B. suzhenae* and *B. bungaroides* indicates the existence of β-BGT in the venoms of these two species.

LAAO, a flavoprotein homodimer [57,58], was present in both venoms and contributes to cytotoxicity by generating reactive oxygen species (ROS) such as H_2_O_2_ during catalysis. These ROS can degrade cell membranes, leading to local symptoms such as swelling, pain, and even necrosis [59,60,61]. In addition to its cytotoxic effects, LAAO modulates platelet aggregation [62] and induces hemorrhage [63] and myonecrosis [64]. *B. suzhenae* venom contained 10.33% (Figure 5), whereas *B. bungaroides* venom had only 4.21% (Figure 5). Compared to other kraits, LAAO content was similar to that reported in *B. fasciatus* venom from Vietnam (7.07%) [26]. In contrast, LAAO levels in *B. multicinctus* [24,25] and *B. sindanus* [28] venoms were much lower (<1%).

The SVMP belongs to the M12 reprolysin subfamily of metalloproteinases. In the venom of *B. suzhenae* and *B. bungaroides*, the SVMPs detected belong to the P-III subclass, which are high-molecular-weight proteins containing a disintegrin-like domain and a cysteine-rich domain following the ADAM metalloprotease domain [65]. SVMPs contribute to local and systemic hemorrhage through various mechanisms, including degradation of basement membranes and extracellular matrix, promotion of apoptosis, inhibition of platelet aggregation, and hydrolysis of collagen, fibrinogen, and induction of leukocyte rolling [65]. Proteomic studies on *B. multicinctus* from Guangxi, China [25], and Vietnam [26] show that SVMP accounts for only 0.05% and 0.78% of the total venom composition, respectively, while *B. sindanus* venom from Pakistan contains 0.45% [28] (Figure 5). In contrast, *B. flaviceps* venom from Malaysia contains a notably higher SVMP proportion of 12.85% [29]. In our study, the significant quantities of LAAO and SVMP detected in *B. suzhenae* venom may explain the intense pain observed at the bite site, as reported in clinical cases [66]. Compared to the venom composition of *B. multicinctus* [24,25,26], the higher levels of LAAO and SVMP in *B. suzhenae* could contribute to its increased toxicity.

AChE is another minor venom protein that promotes the degradation of the neurotransmitter acetylcholine, potentially acting synergistically with paralytic neurotoxins to disrupt acetylcholine metabolism at neuromuscular junctions, leading to muscle paralysis [67]. As shown in Figure 5, the AChE content in the venom of *B. suzhenae* and *B. bungaroides* is 3.72% and 1.58%, respectively. In contrast, proteomic studies of *B. multicinctus* venom from Taiwan [24], Guangxi, China [25] and Vietnam [26] report AChE levels of 0.53%, 0.02%, and 1.10%, respectively. Notably, AChE content reaches approximately 5% in *B. fasciatus* and *B. candidus* from Malaysia and Vietnam [9,26].

In this study, a comprehensive comparison was conducted regarding the relative abundance of toxins among various species of the *Bungarus*. Specifically, *B. multicinctus* (Guangdong [24], Taiwan [24], and Vietnam [26]), *B. fasciatus* (Vietnam [26]), *B. sindanus* (Pakistan [28]), and *B. flaviceps* (Malaysia [29]) were quantitatively analyzed using the extracted ion chromatogram (XIC) method, while *B. caeruleus* (India [27]) was quantified through the spectral counting approach. Spectral counting is based on the acquired MS2 spectra (normalized spectral abundance factor, NSAF), and is involves calculating the ratio of the spectral count of a specific protein to its length [68]. This strategy mitigates technical deviations, making the samples more comparable overall and thereby enhancing the reliability of subsequent statistical analyses. However, spectral counting is unable to quantify low-abundance variations, which restricts the accurate quantification of these proteins [69]. Specific ions (XIC) extracted from LC-MS measurements are quantified by computing the area under the curve or the peak height of each peptide eluted from the LC column within the expected retention time [70]. Compared to spectral counting, XIC demands more computationally intensive data analysis and exhibits higher accuracy. The proteomics quantification method adopted in our research is based on data-independent acquisition (DIA), carried out at the MS2 level by extracting fragment ion chromatograms. Compared to MS1-based extracted ion chromatograms, fragment ion chromatograms are less susceptible to interference and can achieve higher accuracy [71]. Owing to the disparity in the quantification methodologies, the precision of this comparison could potentially be constrained by the existence of low-abundance proteins within the venom; however, it exerts a relatively minor influence on the comparative study of the predominant toxins.

Overall, based on the proteomic data, it is plausible that the presynaptic neurotoxin β-BGT in *B. suzhenae* venom inhibits neurotransmitter release, impairing neuromuscular transmission, while the postsynaptic neurotoxin κ-BGT acts as a high-affinity nAChR antagonist, contributing to neuromuscular blockage and muscle paralysis. The combined action of presynaptic and postsynaptic toxins is likely a key factor in causing neuromuscular paralysis. The biological activity of 3-FTxs such as MTLP, SNX, and WNX remains poorly understood, but they may play secondary roles in neurotoxicity. Additionally, the higher levels of LAAO, SVMP, and AChE in *B. suzhenae* venom increase the complexity of its toxicity, while in *B. bungaroides* venom, β-BGT is the primary neurotoxin responsible for neuromuscular paralysis. It is well established that venom toxicity is closely linked to the types and amounts of toxins present. Given that WNX accounts for over 90% of the total 3-FTx content, it is hypothesized that the overall toxicity of *B. bungaroides* venom may be significantly reduced.

To verify the accuracy of the proteomic results, key enzymatic components identified in the venom were subjected to activity assays. The results revealed biochemical activity differences between *B. suzhenae* and *B. bungaroides* venoms (Figure 6 and Figure 7). PLA_2_ hydrolyzes phospholipids in membranes, disrupting cell membrane structure and function, leading to hemolysis. The lysophospholipids produced during catalysis exhibit high surface activity, inserting into red blood cell membranes, altering their permeability and stability, and promoting hemolysis. Previous studies have shown that basic PLA_2_ exhibits strong hemolytic activity but weak catalytic activity, while acidic PLA_2_ has higher catalytic activity without hemolytic potential [72]. In our study, *B. bungaroides* venom exhibited stronger catalytic and hemolytic activity compared to *B. suzhenae* venom. The higher content of basic PLA_2_ in *B. bungaroides* venom may explain its elevated hemolytic activity, while the similar levels of acidic PLA_2_ in both species indicate that the higher enzymatic activity observed in *B. bungaroides* may be due to its greater abundance of basic PLA_2_.

LAAO contributes to venom toxicity through its pharmacological effects, such as cytotoxicity, hemorrhage, apoptosis induction, and inhibition of platelet aggregation. LAAO exhibits high specificity for L-amino acids. In our study, the venom of *B. suzhenae* exhibited stronger oxidative deamination of amino acids (Figure 6B), suggesting a greater toxic complexity compared to *B. bungaroides*.

Proteolytic and fibrinogenolytic activities are typically mediated by SVMP and SVSP toxins. In our study, both venoms exhibited low or negligible proteolytic activity (Figure 6C), but *B. suzhenae* venom demonstrated significant fibrinogenolytic activity, with a stronger hydrolytic capacity than *B. bungaroides* (Figure 6D). This activity was completely inhibited by EDTA, suggesting metal ion dependency. Our findings are consistent with those of Laxme, who reported low or negligible proteolytic activity for SVMP in *B. caeruleus*, *B. sindanus*, and *B. fasciatus* venoms [73]. These results suggest that SVMP in Bungarus species may lack proteolytic activity, although further isolation and characterization are needed.

In conclusion, based on current research on *Bungarus* species (Table 2), *B. suzhenae* appears to exhibit the highest toxicity. According to our proteomic findings, neurotoxic components such as κ-BGT and β-BGT, which are highly lethal, are present in *B. suzhenae* venom, although their relative abundance is lower compared to *B. multicinctus*. The presence of toxins such as PLA_2_, LAAO, and SVMP contributes to the complexity of *B. suzhenae* venom, which may explain why its LD_50_ is slightly lower than that of *B. multicinctus*. In contrast, *B. bungaroides* venom contains a large amount of weak neurotoxins (31.55%), accounting for over 90% of the total 3-FTx content. The estimated lethality of WNX ranges from 5 to 80 μg/g, further supporting the hypothesis that the high proportion of WNX may reduce the overall toxicity of *B. bungaroides* venom.

## 4. Conclusions

In this study, we employed mass spectrometry-based proteomic analysis to investigate the venom composition of *B. suzhenae* and *B. bungaroides*. In *B. suzhenae* venom, PLA_2_ was the most abundant component (40.29%), followed by 3-FTx (25.84%) and LAAO (10.33%). In *B. bungaroides* venom, PLA_2_, 3-FTx, and KUN were the main toxin components, accounting for 37.91%, 33.87%, and 16.6%, respectively. Enzyme activity assays corroborated the proteomic findings, providing insight into the toxicity of *B. suzhenae* and *B. bungaroides* venoms, as reflected by their LD_50_ values. Comparative analysis with proteomic studies of other species within the genus revealed significant differences in the types and abundance of major toxins between species. These variations, observed both within and between species, are largely influenced by factors such as geographical distribution, sex, ecology, and dietary preferences. The proteomic exploration of *B. suzhenae* and *B. bungaroides* venoms in this study provides a foundation for the formulation and application of traditional and next-generation antivenoms, as well as for clinical treatment of envenomations caused by these species.

## 5. Materials and Methods

### 5.1. Snake Venom

The venom of *B. suzhenae* was collected from three male specimens originally caught in Yingjiang County, Dehong Prefecture, Yunnan Province, China, while the venom of *B. bungaroides* was sourced from one female and two male specimens collected in Motuo County, Tibet, China. These species were captured in the wild and subsequently reared on farms. Fresh venom was manually extracted and stored in collection tubes, followed by centrifugation at 10,000 rpm for 15 min at 4 °C to remove impurities. The venom was then lyophilized and stored at −20 °C until subsequent utilization.

### 5.2. Venom Proteomics

#### 5.2.1. Protein Digestion and Peptide Desalting

A total of 100 μg of dried venom protein powder was dissolved in 10 mM dithiothreitol (DTT; 1064272-Adamas-beta, Shanghai, China) and incubated at 55 °C for 30 min. The solution was subsequently cooled on ice to room temperature and then underwent alkylation with 55 mM iodoacetamide (IAA; 16125-Sigma, St. Louis, MO, USA) for 15 min in the dark at room temperature. Protein precipitation was achieved by adding six volumes of acetone and incubating at −20 °C for at least 4 h. The precipitate was harvested by centrifugation at 8000 rpm for 10 min at 4 °C. After evaporating acetone for 2–3 min, the pellet was resuspended in 100 μL of ammonium bicarbonate (50 mM, H_4_HCO_3_) and digested overnight at 37 °C with trypsin-TPCK (Promega/V5280, Madison, WI, USA) at a concentration of 1 mg/mL (0.1%).

Peptides were subjected to desalination using a SOLA™ SPE 96-well plate. The columns were activated thrice with 200 μL methanol (60209-001-ThermoFisher, Waltham, MA, USA) and equilibrated threefold with 200 μL water containing 0.1% formic acid. After loading 500 μL of the sample, the flow rate was adjusted to 1 mL/min under vacuum. The columns were washed three times with 200 μL of 0.1% formic acid in water, followed by elution of the peptides with 150 μL of 50% acetonitrile in water containing 0.1% formic acid, repeated three times. The eluates were pooled (450 μL total) and vacuum-dried.

#### 5.2.2. Mass Spectrometry and Data Analysis

Prior to mass spectrometry (MS) analysis, three samples from each species were mixed with iRT (internal retention time) standards at a 1:20 ratio. Identical amounts of peptides from each digested sample were separated using an EASY-nLC 1200 system (ThermoFisher) with a C18 analytical column (15 cm × 75 μm ID, 1.6 μm C18, ionopticks). The mobile phase A was constituted by 0.1% formic acid in water, and mobile phase B contained 0.1% formic acid in acetonitrile (ACN), with a flow rate of 400 nL/min. The gradient was as follows: 5–22% B for 0–20 min, 22–37% B for 20–24 min, 37–80% B for 24–27 min, and 80% B for 27–30 min. Peptides were analyzed by a TIMS TOF Pro mass spectrometer (Bruker, Massachusetts, USA) under the following conditions: capillary voltage 1.4 kV, drying gas temperature 180 °C, drying gas flow rate 3.0 L/min, MS scan range 100–1700 m/z, ion mobility range 0.7–1.3 Vs/cm^2^, and collision energy 20–59 eV.

#### 5.2.3. Identification and Quantification of Proteins

The DIA raw data were processed via Spectronaut Pulsar™ 18.4 (Biognosys, Schlieren, Switzerland). The data were subjected to a search against the Elapidae protein database from Uniprot. The parameters encompassed a precursor mass threshold of 0.01 ppm and a protein mass threshold of 0.01 Da. Carbamidomethylation of cysteine was stipulated as a fixed modification; however, oxidation (M) and acetylation (N-term) were defined as variable modifications, allowing a maximum of two missed cleavage sites per peptide. Proteins were identified based on at least one unique peptide. Redundant peptides were excluded based on peptide Morpheus scores, and homologous proteins were aligned to verify the accuracy of identifications. Quantification was accomplished by extracting fragment ion chromatograms at the MS2 level.

### 5.3. Biochemical Characterization

#### 5.3.1. PLA_2_ Activity

PLA_2_ activity was measured in accordance with the method described by Haberman [74]. Egg yolk was mixed with 0.9% NaCl solution at a ratio of 1:3, followed by centrifugation at 3000 rpm for 5 min (IKA^®^VORTEX, Guangzhou, China). The supernatant was used as the substrate. Agarose (0.6 g) (SCR/10000561-250g, Shanghai, China) was dissolved in 20 mL NaAC buffer (50 mM, pH 7.5) (SCR/10018818-500g, Shanghai, China) and microwaved until fully dissolved. After cooling to 50 °C, 4% egg yolk substrate and 2% CaCl_2_ were added. The mixture was poured into Petri dishes, cooled to solidify, and holes were punched. Samples (10–50 μg of venom) were added, and plates were incubated at 37 °C for 15 h. The minimum venom dose that produced a 20 mm clear zone (MPD) was used as the measure of activity.

#### 5.3.2. LAAO Activity

LAAO activity was measured in accordance with the methodology proposed by Tasoulis [75]. Venom samples (10–50 μg) were dissolved in 50 mM Tris-HCl buffer (pH 7.5) (Sigma/T5941-100g, St. Louis, MO, USA) and incubated at 37 °C for 10 min. A reaction mixture containing 5 mM L-leucine, 50 nM Tris-HCl, 5 IU/ml horseradish peroxidase (Aladdin/P105526-5mg, Shanghai, China), and 2 mM o-phenylenediamine hydrochloride (Sigma/P239385g, St. Louis, MO, USA) was added, and the solution was incubated for 60 min at 37 °C. The reaction was halted by the addition of 50 μL of 2 M H_2_SO_4_, and the absorbance was measured at 492 nm.

#### 5.3.3. SVMP Activity

SVMP activity was detected with modifications from the method by R.R. Senji Laxme [76]. A total of 50 μL substrate solution (50 mM Tris-HCl, pH 7.5, with 1% azocasein) was mixed with 10–50 μg of venom in 10 μL ddH_2_O. Following incubation at 37 °C for 60 min, the reaction was halted by adding 150 μL of a 5% trichloroacetic acid (TCA) solution (Sigma/T6508-100ML, St. Louis, MO, USA) and allowing it to incubate for an additional 30 min. The supernatant was then collected and treated with 50 μL of a 2M NaOH solution. Absorbance readings were taken at a wavelength of 440 nm.

#### 5.3.4. Fibrinogen Degradation Assay

Venom’s fibrinogenolytic activity was assessed using SDS-PAGE [77]. Briefly, 20 μg of human fibrinogen in phosphate-buffered saline (PBS, pH 7.4) was incubated with 2 μg of crude venom at 37 °C for 60 min. Post-incubation, loading buffer (1 M Tris-HCl, pH 6.8; 50% glycerol; 0.5% bromophenol blue; 10% SDS; and 20% β-mercaptoethanol) was incorporated into the samples, which were subsequently heated at 100 °C for 10 min. Electrophoresis was conducted on a 12.5% SDS-PAGE gel, and fibrinogen degradation products were compared to untreated controls. For inhibition studies, the venom was pre-incubated with EDTA to assess the role of SVMP.

#### 5.3.5. Hemolysis Assay

The direct hemolysis assay was conducted using two methods: one with phospholipid and one without [78]. Fresh RBCs were isolated from mouse plasma, followed by washing with 0.9% NaCl to remove residual proteins. This washing process was repeated three times at 5000 rpm for 10 min each time. After the final wash, the supernatant was dispensed, and the RBCs were resuspended in phosphate-buffered saline (PBS, pH 7.4) to prepare an RBC suspension. The RBC suspension was mixed with venom at a 1:10 ratio and incubated at 37 °C for 24 h. Hemolytic activity was determined by measuring the absorbance of the supernatant at 595 nm using a spectrophotometer. Triton X (1%) was utilized as the positive control, representing 100% activity, and the relative hemolytic activity of the venom was computed accordingly.

For the phospholipid-containing method [79], egg yolk was mixed with 0.85% NaCl solution at a 1:3 volume ratio. The mixture was subjected to centrifugation at 3000 rpm for 5 min, and the supernatant was collected as the egg yolk substrate. Agarose (0.6 g) was dissolved in 60 mL of NaAc solution (50 mM, pH 7.5) using a microwave. Once the solution cooled to 50 °C, 4% egg yolk substrate, 2% CaCl_2_ solution, and the RBC suspension were added, mixed thoroughly, and poured into a glass Petri dish. After the agar solidified, wells were punched for sample application. The plates were incubated at 37 °C in a constant temperature and humidity incubator for 12 h. The diameter of the clear zones was measured, and the minimum venom dose required to induce a 20 mm clear zone (MPD) was used as a measure of hemolytic potency.

### 5.4. LD_50_ Test

Male KM mice were used for the LD_50_ test, which weighed 18–22 g. After a 5–7-day acclimatization period, the mice were randomly partitioned into groups, with six mice per concentration group. Each group received a tail vein injection of 100 μL venom from *B. suzhenae* or *B. bungaroides*, dissolved in sterile saline. The control group received an equivalent volume of saline. Mortality was documented over a 24-h period, and the LD50 was calculated using the Spearman–Karber method.

### 5.5. Statistical Analysis

All experiments were performed in triplicate. The data were presented as means ± standard error (SE), based on three independent determinations for each experiment. Statistical analyses were implemented using GraphPad Prism 9.0.0 software. The discrepancies among mean values were analyzed by analysis of variance (ANOVA), with a 95% confidence interval. Statistical significance was defined as *p* < 0.05.

## Figures and Tables

**Table 1:** Identification of proteins in the venoms of *B. suzhenae* and *B. bungaroides* using liquid chromatography–tandem mass spectrometry (LC-MS/MS).

Protein Family	Accession	Description	Source Organism	Expression Quantity	
				*B. suzhenae*	*B. bungaroides*
3-FTx	O12962	Kappa-5-bungarotoxin	*B. multicinctus*	7.02%	<0.01%
	P43445	Short neurotoxin homolog	*B. multicinctus*	0.09%	0.19%
	Q9YGI8	Short neurotoxin homolog NTL4	*B. multicinctus*	1.00%	1.21%
	P01473	Cytotoxin homolog 3	*N. melanoleuca*	——	0.08%
	Q7ZT13	Neurotoxin-like protein pMD18-NTL1/2/4/5	*B. multicinctus*	2.40%	0.01%
	Q53B61	Weak neurotoxin OH-72	*O. hannah*	——	0.91%
	P01399	Weak toxin CM-13b	*N. annulifera*	——	<0.01%
	P01400	Weak toxin S4C11	*N. melanoleuca*	7.32%	28.89%
	Q8AY50	Weak toxin 2	*B. candidus*	0.88%	<0.01%
	A2CKF6	Neurotoxin 3FTx-8a	*B. fasciatus*	1.19%	1.75%
	Q8JFX7	Muscarinic toxin BM14	*B. multicinctus*	3.20%	0.01%
	D5J9Q3	Muscarinic toxin-like protein	*B. flaviceps*	——	0.15%
	P81783	Candoxin	*B. candidus*	0.22%	0.64%
	Q70WS8	Neurotoxin BM10-1-like	*B. multicinctus*	0.13%	——
	Q9YGJ0	Gamma-bungarotoxin	*B. multicinctus*	1.15%	0.01%
	P81782	Bucandin	*B. candidus*	1.22%	0.01%
	R4FIT5	3FTx-Pse-89	*P.modesta*	——	0.02%
	A0A898IN56	Three-finger toxin	*C. bivirgatus*	<0.01%	——
	A0A898IN96	Three-finger toxin	*C. bivirgatus*	0.03%	——
PLA2	P00619	Acidic phospholipase A_2_ beta-bungarotoxin A3 chain	*B. multicinctus*	1.88%	0.05%
	P00618	Basic phospholipase A_2_ beta-bungarotoxin A2 chain	*B. multicinctus*	3.93%	0.02%
	Q75S51	Basic phospholipase A_2_ beta-bungarotoxin A4 chain	*B. candidus*	1.53%	0.01%
	Q8QFW4	Basic phospholipase A_2_ beta-bungarotoxin A1 chain	*B. caeruleus*	0.01%	——
	Q9PTA6	Basic phospholipase A_2_ beta-bungarotoxin A-AL3 chain	*B. multicinctus*	0.07%	——
	D5J9R7	Beta-bungarotoxin a chain isoform 1	*B. flaviceps*	1.45%	3.41%
	Q8AXW2	Beta-bungarotoxin A8 chain	*B. multicinctus*	——	0.11%
	P00606	Acidic phospholipase A_2_	*B. multicinctus*	1.93%	0.03%
	P0C551	Acidic phospholipase A_2_ KBf-grIB	*B. fasciatus*	0.38%	0.53%
	P29601	Acidic phospholipase A_2_ homolog	*B. fasciatus*	——	0.71%
	Q45Z26	Acidic phospholipase A_2_ 5	*T. carinatus*	0.46%	0.29%
	Q75S48	Acidic phospholipase A_2_ 1	*B. candidus*	0.73%	0.01%
	Q802I1	Acidic phospholipase A_2_ Bc-PL	*B. candidus*	6.40%	0.01%
	Q9PUH8	Acidic phospholipase A_2_ S3-24	*A. superbus*	0.38%	0.70%
	Q9PUH9	Acidic phospholipase A_2_ S9-53F	*A. superbus*	<0.01%	2.44%
	P10116	Basic phospholipase A_2_ 2	*L. colubrina*	1.38%	0.17%
	P14556	Basic phospholipase A_2_ nigexine	*N. pallida*	——	0.15%
	P60043	Basic phospholipase A_2_ 1	*N. sagittifera*	0.02%	<0.01%
	P81236	Basic phospholipase A_2_ acanthin-1	*A. antarcticus*	<0.01%	0.01%
	P84472	Basic phospholipase A_2_ T1-2 A chain	*B. candidus*	<0.01%	——
	P84474	Basic phospholipase A_2_ T2 A chain	*B. candidus*	12.54%	0.07%
	Q6SLM1	Basic phospholipase A_2_ 2	*B. caeruleus*	3.00%	0.02%
	Q9DF52	Basic phospholipase A_2_ KPA2	*B. caeruleus*	1.43%	14.79%
	P00595	Basic phospholipase A_2_ DE-1	*H. haemachatus*	——	0.03%
	A8S6C5	Phosphatidylcholine 2-acylhydrolase	*A. superbus*	0.01%	1.58%
	P59066	Phospholipase A_2_	*A. superbus*	——	0.01%
	B2BRS5	Phosphatidylcholine 2-acylhydrolase	*A. labialis*	0.01%	——
	B2KNL4	phospholipase A_2_ BF-40	*B. fasciatus*	0.09%	<0.01%
	R4G2E0	phospholipase A_2_	*B. roperi*	0.01%	——
	A6MFM6	PLA_2_-7	*D. vestigiata*	0.11%	——
	A6MFM7	PLA_2_-2	*C. nigrescens*	——	0.01%
	A6MFM9	PLA_2_-4	*C. nigrescens*	0.04%	——
	R4G2E6	PLA_2_-Cac-18	*C. squamulosus*	——	0.01%
	A0A0F7YYZ6	phospholipase A_2_	*M. fulvius*	0.05%	0.02%
	A0A2D4IRB3	phospholipase A_2_	*M. lemniscatus*	0.96%	——
	A0A2D4IRN4	phospholipase A_2_	*M. lemniscatus*	0.30%	<0.01%
	A0A2D4L945	phospholipase A_2_	*M. paraensis*	1.03%	——
	A0A2D4NPI3	phospholipase A_2_	*M. spixii*	0.01%	0.05%
	A0A2D4Q1F6	Phospholipase A_2_ domain-containing protein	*M. surinamensis*	0.15%	——
	A0A898INR6	phospholipase A_2_	*C. bivirgatus*	<0.01%	3.28%
	A0A898IPP8	phospholipase A_2_	*C. bivirgatus*	0.01%	9.38%
PLB	V8ND68	Phospholipase B-like	*O. hannah*	0.11%	0.10%
	A0A8C7E4Y6	Phospholipase B-like	*N. naja*	0.01%	0.01%
	A0A8C5RQ29	Phospholipase B-like	*L. laticaudata*	0.09%	0.10%
CRISP	A6MFK9	Cysteine-rich venom protein	*D. vestigiata*	0.32%	<0.01%
	Q7ZT98	Cysteine-rich venom protein ophanin	*O. hannah*	<0.01%	0.24%
	F2Q6G2	Cysteine-rich seceretory protein Bc-CRPb	*B. candidus*	2.55%	0.03%
	R4G2Q3	CRiSP-Bra-2	*B. roperi*	0.01%	<0.01%
	P0DL15	Cysteine-rich venom protein annuliferin-b	*N. naja*	——	0.20%
	C1JZW4	Opharin	*O. hannah*	0.01%	0.27%
	A0A670YAR1	ShKT domain-containing protein	*P. textilis*	——	0.09%
SVMP	A8QL49	Zinc metalloproteinase-disintegrin-like BmMP	*B. multicinctus*	0.65%	0.77%
	A8QL59	Zinc metalloproteinase-disintegrin-like NaMP	*N. atra*	<0.01%	<0.01%
	D3TTC1	Zinc metalloproteinase-disintegrin-like kaouthiagin-like	*N. atra*	<0.01%	0.12%
	P0DJ43	Zinc metalloproteinase-disintegrin-like mikarin	*M. ikaheca*	0.01%	——
	Q10749	Snake venom metalloproteinase-disintegrin-like mocarhagin	*N. mossambica*	0.33%	0.27%
	R4FJY3	SVMP-Hop-63	*H. bungaroides*	<0.01%	<0.01%
	R4FJZ4	SVMP-Hop-15	*H. bungaroides*	0.02%	0.14%
	R4G2D3	SVMP-Aca-4	*A. wellsi*	<0.01%	0.07%
	A0A194AS47	Metalloproteinase type III 2	*M. tener*	4.92%	0.08%
	A0A898ILL7	Snake venom metalloprotease	*C. bivirgatus*	0.03%	0.05%
	A0A898IKT8	Snake venom metalloprotease	*C. bivirgatus*	0.18%	0.37%
SVSP	A8QL57	Snake venom serine protease BmSP	*B. multicinctus*	0.01%	——
LAAO	A8QL51	L-amino-acid oxidase	*B. multicinctus*	9.29%	1.97%
	A8QL52	L-amino-acid oxidase	*B. fasciatus*	0.94%	1.37%
	A0A2D4KMW9	L-amino-acid oxidase	*M. paraensis*	<0.01%	0.02%
	A0A2U8QNU6	L-amino-acid oxidase	*M. mipartitus*	0.04%	0.04%
	A0A2D4KYN2	L-amino-acid oxidase	*M. paraensis*	<0.01%	0.80%
	A0A8C5S978	L-amino-acid oxidase	*L. laticaudata*	0.05%	——
	A0A8C6V9N4	L-amino-acid oxidase	*N. naja*	——	0.01%
CTL	A1XXJ9	C-type lectin BML-2	*B. multicinctus*	0.01%	0.01%
	Q90WI6	C-type lectin BML-1	*B. multicinctus*	0.05%	0.09%
	V8P038	C-type lectin domain-containing protein	*O. hannah*	<0.01%	0.01%
	A0A8C5SBY4	C-type lectin domain-containing protein	*L. laticaudata*	——	0.01%
KUN	B4ESA4	Kunitz-type serine protease inhibitor PILP-3	*B. multicinctus*	0.84%	0.05%
	Q8AY42	Kunitz-type serine protease inhibitor B	*B. candidus*	0.90%	——
	P25660	Kunitz-type serine protease inhibitor IX	*B. fasciatus*	——	0.42%
	Q0PL65	Kunitz-type serine protease inhibitor homolog beta-bungarotoxin B5-B chain	*B. multicinctus*	0.30%	0.00%
	Q75S49	Kunitz-type serine protease inhibitor homolog beta-bungarotoxin B4 chain	*B. candidus*	0.06%	——
	Q75S50	Kunitz-type serine protease inhibitor homolog beta-bungarotoxin B3 chain	*B. candidus*	4.15%	0.01%
	P00989	Kunitz-type serine protease inhibitor homolog beta-bungarotoxin B2 chain	*B. multicinctus*	0.22%	<0.01%
	D5J9R0	Beta-bungarotoxin b chain isoform 1	*B. flaviceps*	3.02%	16.12%
NP	D9IX98	Natriuretic peptide Oh-NP	*O. hannah*	0.20%	——
	A0A194APA7	C-type natriuretic peptide	*M. tener*	0.03%	<0.01%
CYS	E3P6N4	Cystatin	*P. textilis*	<0.01%	0.04%
	P81714	Cystatin	*N. atra*	0.01%	0.10%
NGF	P34128	Venom nerve growth factor	*B. multicinctus*	0.30%	1.19%
AChE	Q92035	Acetylcholinesterase	*B. fasciatus*	3.72%	1.58%
PI	Q78CF8	Phospholipase A2 inhibitor	*O. scutellatus*	<0.01%	0.01%
	V8N5K6	Phospholipase A2 inhibitor	*O. hannah*	——	<0.01%
	P0DUK6	Phospholipase A2 inhibitor gamma subunit B	*L. semifasciata*	<0.01%	0.01%
	A0A2D4JM54	BPTI/Kunitz inhibitor domain-containing protein	*M. lemniscatus*	<0.01%	——
PDE	A0A2D0TC04	Venom phosphodiesterase	*N. atra*	0.01%	0.10%
	A0A0F7YYZ8	Phosphodiesterase	*M. fulvius*	<0.01%	——
5′-NT	A0A0F7YZM6	5′-nucleotidase	*M. ulvius*	<0.01%	0.01%
	A0A670YK89	5′-nucleotidase	*P. textilis*	0.02%	——
	A0A8C6XAL5	5′-nucleotidase	*N. naja*	0.09%	0.46%
	A0A8C7DVT3	5′-nucleotidase	*N. naja*	0.03%	0.17%
VESP	A0A194AR88	Vespryn	*M. tener*	——	0.01%
	A0A2D4NZ30	B30.2/SPRY domain-containing protein	*M. surinamensis*	<0.01%	0.65%
HAase	A0A6J1U3D2	Hyaluronidase	*N. scutatus*	0.24%	0.01%
	A0A898INC5	Hyaluronidase	*C. bivirgatus*	0.09%	<0.01%

**Table 2:** Comparison of LD_50_ values for *B. suzhenae* and *B. bungaroides* venoms with other species of the *Bungarus* genus.

Species	LD_50_ (μg/g)	Injection Strategy	Sources
*B. suzhenae*	0.0133 (0.01–0.015)	*i.v.*	This work
*B. bungaroides*	0.752 (0.075–0.8)	*i.v.*	This work
*B. multicinctus* (Guangdong)	0.027 (0.026–0.028)	*i.v.*	[24]
*B. multicinctus* (Taiwan)	0.087 (0.084–0.091)	*i.v.*	[24]
	0.10 (0.08–0.12)	*s.c.*	[30]
*B. multicinctus* (Guangxi)	0.016 (0.013–0.019)	*i.p.*	[25]
*B. sindanus*	0.04 (0.035–0.045)	*i.v.*	[28]
	0.15 (0.14–0.16)	*s.c.*	[30]
*B. candidus*	0.11 (0.07–0.17)	*i.v.*	[31]
	0.09 (0.06–0.14)	*i.v.*	[32]
	0.50 (0.45–0.56)	*s.c.*	[30]
*B. fasciatus*	0.45 (0.30–0.68)	*i.v.*	[31]
	1.20 (1.13–1.27)	*s.c.*	[30]
*B. flaviceps*	0.25 (0.20–0.31)	*i.v.*	[29]
*B. caeruleus*	0.10 (0.081–0.123)	*i.v.*	[28]
	0.50 (0.45–0.56)	*s.c.*	[30]

*i.v.*: intravenous; *i.p.*: intraperitoneal; *s.c.*: subcutaneous.

## Data Availability

The data presented in this study are available in this article and supplementary materials.

## Supplementary Materials

The following supporting information can be downloaded at: https://www.mdpi.com/article/10.3390/toxins16110494/s1. Table S1: Protein matches that were obtained by tandem MS/MS of tryptic peptides from captive *B. suzhenae* and *B. bungaroides* venom. Figrue S1: PLA_2_ activity test of the original gel, Figure S2: PLA_2_ hemolysis activity test of the original gel.

## References

[1] WHO Snakebite-Envenoming. https://www.who.int/zh/news-room/fact-sheets/detail/snakebite-envenoming.

[2] Gutiérrez J.M., Calvete J.J., Habib A.G., Harrison R.A., Williams D.J., Warrell D.A. (2017). Snakebite envenoming. Nat. Rev. Dis. Primers.

[3] Winkel K., Kasturiratne A., Wickremasinghe A.R., de Silva N., Gunawardena N.K., Pathmeswaran A., Premaratna R., Savioli L., Lalloo D.G., de Silva H.J. (2008). The Global Burden of Snakebite: A Literature Analysis and Modelling Based on Regional Estimates of Envenoming and Deaths. PLoS Med..

[4] Chippaux J.-P. (2017). Snakebite envenomation turns again into a neglected tropical disease!. J. Venom. Anim. Toxins Incl. Trop. Dis..

[5] Uetz P., Hosek J. The Reptile Database. https://reptile-database.reptarium.cz/.

[6] Rusmili M.R.A., Yee T.T., Mustafa M.R., Hodgson W.C., Othman I. (2014). Isolation and characterization of a presynaptic neurotoxin, P-elapitoxin-Bf1a from Malaysian Bungarus fasciatus venom. Biochem. Pharmacol..

[7] Rusmili M.R.A., Tee T.Y., Mustafa M.R., Othman I., Hodgson W.C. (2014). Isolation and characterization of α-elapitoxin-Bf1b, a postsynaptic neurotoxin from Malaysian Bungarus fasciatus venom. Biochem. Pharmacol..

[8] Rossetto O., Morbiato L., Caccin P., Rigoni M., Montecucco C. (2006). Presynaptic enzymatic neurotoxins. J. Neurochem..

[9] Rusmili M.R.A., Yee T.T., Mustafa M.R., Hodgson W.C., Othman I. (2014). Proteomic characterization and comparison of Malaysian Bungarus candidus and Bungarus fasciatus venoms. J. Proteom..

[10] WHO Guidelines for the Management of Snakebites. https://www.who.int/publications/i/item/9789290225300.

[11] Tan N.H., Tan K.Y., Tan C.H. (2021). Snakebite in Southeast Asia: Envenomation and Clinical Management.

[12] Chen Z.-N., Shi S.-C., Vogel G., Ding L., Shi J.-S. (2021). Multiple lines of evidence reveal a new species of Krait (Squamata, Elapidae, Bungarus) from Southwestern China and Northern Myanmar. ZooKeys.

[13] Kuch U., Kizirian D., Truong N.Q., Lawson R., Donnelly M.A., Mebs D., Lannoo M.J. (2005). A New Species of Krait (Squamata: Elapidae) from the Red River System of Northern Vietnam. Copeia.

[14] Slowinski J.B. (1994). A Phylogenetic Analysis of Bungarus (Elapidae) Based on Morphological Characters. J. Herpetol..

[15] Nie X., Chen Q., Wang C., Huang W., Lai R., Lu Q., He Q., Yu X. (2022). Venom Variation of Neonate and Adult Chinese Cobras in Captivity Concerning Their Foraging Strategies. Toxins.

[16] Tan C.H., Palasuberniam P., Tan K.Y. (2021). Snake Venom Proteomics, Immunoreactivity and Toxicity Neutralization Studies for the Asiatic Mountain Pit Vipers, Ovophis convictus, Ovophis tonkinensis, and Hime Habu, Ovophis okinavensis. Toxins.

[17] Jaglan A., Bhatia S., Martin G., Sunagar K. (2023). The Royal Armoury: Venomics and antivenomics of king cobra (*Ophiophagus hannah*) from the Indian Western Ghats. Int. J. Biol. Macromol..

[18] Chanda A., Patra A., Kalita B., Mukherjee A.K. (2018). Proteomics analysis to compare the venom composition between *Naja naja* and *Naja kaouthia* from the same geographical location of eastern India: Correlation with pathophysiology of envenomation and immunological cross-reactivity towards commercial polyantivenom. Expert Rev. Proteom..

[19] Mora-Obando D., Salazar-Valenzuela D., Pla D., Lomonte B., Guerrero-Vargas J.A., Ayerbe S., Gibbs H.L., Calvete J.J. (2020). Venom variation in Bothrops asper lineages from North-Western South America. J. Proteom..

[20] Nie X., He Q., Zhou B., Huang D., Chen J., Chen Q., Yang S., Yu X. (2021). Exploring the five-paced viper (*Deinagkistrodon acutus*) venom proteome by integrating a combinatorial peptide ligand library approach with shotgun LC-MS/MS. J. Venom. Anim. Toxins Incl. Trop. Dis..

[21] Rodríguez-Vargas A., Franco-Vásquez A.M., Bolívar-Barbosa J.A., Vega N., Reyes-Montaño E., Arreguín-Espinosa R., Carbajal-Saucedo A., Angarita-Sierra T., Ruiz-Gómez F. (2023). Unveiling the Venom Composition of the Colombian Coral Snakes Micrurus helleri, M. medemi, and M. sangilensis. Toxins.

[22] Ogawa T., Tobishima Y., Kamata S., Matsuda Y., Muramoto K., Hidaka M., Futai E., Kuraishi T., Yokota S., Ohno M. (2021). Focused Proteomics Analysis of Habu Snake (*Protobothrops flavoviridis*) Venom Using Antivenom-Based Affinity Chromatography Reveals Novel Myonecrosis-Enhancing Activity of Thrombin-Like Serine Proteases. Front. Pharmacol..

[23] Islam T., Madhubala D., Mukhopadhyay R., Mukherjee A.K. (2021). Transcriptomic and functional proteomics analyses to unveil the common and unique pathway(s) of neuritogenesis induced by Russell’s viper venom nerve growth factor in rat pheochromocytoma neuronal cells. Expert Rev. Proteom..

[24] Oh A.M.F., Tan K.Y., Tan N.H., Tan C.H. (2021). Proteomics and neutralization of *Bungarus multicinctus* (Many-banded Krait) venom: Intra-specific comparisons between specimens from China and Taiwan. Comp. Biochem. Physiol. Part C Toxicol. Pharmacol..

[25] Shan L.-L., Gao J.-F., Zhang Y.-X., Shen S.-S., He Y., Wang J., Ma X.-M., Ji X. (2016). Proteomic characterization and comparison of venoms from two elapid snakes *(Bungarus multicinctus* and *Naja atra*) from China. J. Proteom..

[26] Ziganshin R.H., Kovalchuk S.I., Arapidi G.P., Starkov V.G., Hoang A.N., Thi Nguyen T.T., Nguyen K.C., Shoibonov B.B., Tsetlin V.I., Utkin Y.N. (2015). Quantitative proteomic analysis of Vietnamese krait venoms: Neurotoxins are the major components in *Bungarus multicinctus* and phospholipases A2 in *Bungarus fasciatus*. Toxicon.

[27] Patra A., Chanda A., Mukherjee A.K. (2019). Quantitative proteomic analysis of venom from Southern India common krait (*Bungarus caeruleus*) and identification of poorly immunogenic toxins by immune-profiling against commercial antivenom. Expert Rev. Proteom..

[28] Oh A.M.F., Tan C.H., Tan K.Y., Quraishi N.H., Tan N.H. (2019). Venom proteome of *Bungarus sindanus* (*Sind krait*) from Pakistan and in vivo cross-neutralization of toxicity using an Indian polyvalent antivenom. J. Proteom..

[29] Tan C.H., Oh A.M.F., Wong K.Y., Liew J.L., Tan N.H., Tan K.Y. (2022). On characterizing the Red-headed Krait (*Bungarus flaviceps*) venom: Decomplexation proteomics, immunoreactivity and toxicity cross-neutralization by hetero-specific antivenoms. Comp. Biochem. Physiol. Part D Genom. Proteom..

[30] Tan C.H., Lingam T.M.C., Tan K.Y. (2022). Varespladib (LY315920) rescued mice from fatal neurotoxicity caused by venoms of five major Asiatic kraits (*Bungarus* spp.) in an experimental envenoming and rescue model. Acta Trop..

[31] Tan C.H., Liew J.L., Tan K.Y., Tan N.H. (2016). Assessing SABU (*Serum Anti Bisa Ular*), the sole Indonesian antivenom: A proteomic analysis and neutralization efficacy study. Sci. Rep..

[32] Gutiérrez J.M., Ratanabanangkoon K., Tan K.Y., Eursakun S., Tan C.H., Simsiriwong P., Pamornsakda T., Wiriyarat W., Klinpayom C., Tan N.H. (2016). A Simple and Novel Strategy for the Production of a Pan-specific Antiserum against Elapid Snakes of Asia. PLOS Neglected Trop. Dis..

[33] Duerrschmidt N., Hagen A., Gaertner C., Wermke A., Nowicki M., Spanel-Borowski K., Stepan H., Mohr F.-W., Dhein S. (2012). Nicotine effects on human endothelial intercellular communication via α4β2 and α3β2 nicotinic acetylcholine receptor subtypes. Naunyn-Schmiedeberg’s Arch. Pharmacol..

[34] Intachai K.C., Chattipakorn S., Chattipakorn N., Shinlapawittayatorn K. (2018). Revisiting the Cardioprotective Effects of Acetylcholine Receptor Activation against Myocardial Ischemia/Reperfusion Injury. Int. J. Mol. Sci..

[35] Grant G.A., Al-Rabiee R., Xu X., Zhang Y. (1997). Critical Interactions at the Dimer Interface of κ-Bungarotoxin, a Neuronal Nicotinic Acetylcholine Receptor Antagonist. Biochemistry.

[36] Chiappinelli V.A., Weaver W.R., McLane K.E., Conti-Fine B.M., Fiordalisi J.J., Grant G.A. (1996). Binding of native κ-neurotoxins and site-directed mutants to nicotinic acetylcholine receptors. Toxicon.

[37] White J., Ranawaka U.K., Lalloo D.G., de Silva H.J. (2013). Neurotoxicity in Snakebite—The Limits of Our Knowledge. PLoS Neglected Trop. Dis..

[38] Kukhtina V.V., Weise C., Muranova T.A., Starkov V.G., Franke P., Hucho F., Wnendt S., Gillen C., Tsetlin V.I., Utkin Y.N. (2003). Muscarinic toxin-like proteins from cobra venom. Eur. J. Biochem..

[39] Munawar A., Ali S.A., Akrem A., Betzel C. (2018). Snake Venom Peptides: Tools of Biodiscovery. Toxins.

[40] Dufton M.J., Hider R.C. (1988). Structure and pharmacology of elapid cytotoxins. Pharmacol. Ther..

[41] Pillai L.V., Ambike D., Husainy S., Khaire A., Captain A., Kuch U. (2012). Severe Neurotoxic Envenoming and Cardiac Complications after the Bite of a ‘Sind Krait’ (*Bungarus* cf. sindanus) in Maharashtra, India. Trop. Med. Health.

[42] Tongpoo A., Sriapha C., Pradoo A., Udomsubpayakul U., Srisuma S., Wananukul W., Trakulsrichai S. (2018). Krait envenomation in Thailand. Ther. Clin. Risk Manag..

[43] Utkin Y.N., Kukhtina V.V., Kryukova E.V., Chiodini F., Bertrand D., Methfessel C., Tsetlin V.I. (2001). “Weak Toxin” from *Naja kaouthia* Is a Nontoxic Antagonist of α7 and Muscle-type Nicotinic Acetylcholine Receptors. J. Biol. Chem..

[44] Lay Poh S., Mourier G., Thai R., Armugam A., Molgó J., Servent D., Jeyaseelan K., Ménez A. (2002). A synthetic weak neurotoxin binds with low affinity to Torpedo and chicken α7 nicotinic acetylcholine receptors. Eur. J. Biochem..

[45] Mordvintsev D.Y., Rodionov D.I., Makarova M.V., Kamensky A.A., Levitskaya N.G., Ogay A.Y., Rzhevsky D.I., Murashev A.N., Tsetlin V.I., Utkin Y.N. (2007). Behavioural Effects in Mice and Intoxication Symptomatology of Weak Neurotoxin from Cobra *Naja kaouthia*. Basic Clin. Pharmacol. Toxicol..

[46] Sampat G.H., Hiremath K., Dodakallanavar J., Patil V.S., Harish D.R., Biradar P., Mahadevamurthy R.K., Barvaliya M., Roy S. (2023). Unraveling snake venom phospholipase A2: An overview of its structure, pharmacology, and inhibitors. Pharmacol. Rep..

[47] Gutiérrez J.M., Lomonte B. (2013). Phospholipases A2: Unveiling the secrets of a functionally versatile group of snake venom toxins. Toxicon.

[48] Aloulou A., Rahier R., Arhab Y., Noiriel A., Abousalham A. (2018). Phospholipases: An Overview. Lipases and Phospholipases.

[49] Vuong N.T., Jackson T.N.W., Wright C.E. (2021). Role of Phospholipases A2 in Vascular Relaxation and Sympatholytic Effects of Five Australian Brown Snake, *Pseudonaja* spp., Venoms in Rat Isolated Tissues. Front. Pharmacol..

[50] Strong P.N., Goerke J., Oberg S.G., Kelly R.B. (1976). beta-Bungarotoxin, a pre-synaptic toxin with enzymatic activity. Proc. Natl. Acad. Sci. USA.

[51] Dufton M.J., Hider R.C. (1983). Classification of phospholipases A2 according to sequence. Evolutionary and pharmacological implications. Eur. J. Biochem..

[52] Schmidt R.R., Betz H., Rehm H. (1988). Inhibition of.beta.-bungarotoxin binding to brain membranes by mast cell degranulating peptide, toxin I, and ethylene glycol bis(.beta.-aminoethyl ether)-N,N,N′,N′-tetraacetic acid. Biochemistry.

[53] Rowan E.G. (2001). What does β-bungarotoxin do at the neuromuscular junction?. Toxicon.

[54] Calvete J.J., Lin B., Zhang J.-R., Lu H.-J., Zhao L., Chen J., Zhang H.-F., Wei X.-S., Zhang L.-Y., Wu X.-B. (2020). Immunoreactivity and neutralization study of Chinese *Bungarus multicinctus* antivenin and lab-prepared anti-bungarotoxin antisera towards purified bungarotoxins and snake venoms. PLOS Neglected Trop. Dis..

[55] Laskowski M., Kato I. (1980). Protein Inhibitors of Proteinases. Annu. Rev. Biochem..

[56] Cardle L., Dufton M. (1997). Foci of amino acid residue conservation in the 3D structures of the Kunitz BPTI proteinase inhibitors: How do variants from snake venom differ?. Protein Eng. Des. Sel..

[57] Izidoro L.F.M., Sobrinho J.C., Mendes M.M., Costa T.R., Grabner A.N., Rodrigues V.M., da Silva S.L., Zanchi F.B., Zuliani J.P., Fernandes C.F.C. (2014). Snake Venom L-Amino Acid Oxidases: Trends in Pharmacology and Biochemistry. BioMed Res. Int..

[58] Pawelek P.D., Cheah J.H., Coulombe R., Macheroux P., Ghisla S., Vrielink A. (2000). The structure of L-amino acid oxidase reveals the substrate trajectory into an enantiomerically conserved active site. EMBO J..

[59] Samel M., Vija H., Rönnholm G., Siigur J., Kalkkinen N., Siigur E. (2006). Isolation and characterization of an apoptotic and platelet aggregation inhibiting l-amino acid oxidase from Vipera berus berus (*common viper*) venom. Biochim. Et Biophys. Acta (BBA)—Proteins Proteom..

[60] Ribeiro P.H., Zuliani J.P., Fernandes C.F.C., Calderon L.A., Stábeli R.G., Nomizo A., Soares A.M. (2016). Mechanism of the cytotoxic effect of l-amino acid oxidase isolated from Bothrops alternatus snake venom. Int. J. Biol. Macromol..

[61] Zhang L., Wei L.-J. (2007). ACTX-8, a cytotoxic l-amino acid oxidase isolated from Agkistrodon acutus snake venom, induces apoptosis in Hela cervical cancer cells. Life Sci..

[62] de Queiroz M.R., de Sousa B.B., da Cunha Pereira D.F., Mamede C.C.N., Matias M.S., de Morais N.C.G., de Oliveira Costa J., de Oliveira F. (2017). The role of platelets in hemostasis and the effects of snake venom toxins on platelet function. Toxicon.

[63] Ciscotto P., Machado de Avila R.A., Coelho E.A.F., Oliveira J., Diniz C.G., Farías L.M., de Carvalho M.A.R., Maria W.S., Sanchez E.F., Borges A. (2009). Antigenic, microbicidal and antiparasitic properties of an l-amino acid oxidase isolated from Bothrops jararaca snake venom. Toxicon.

[64] Wei J.-F., Yang H.-W., Wei X.-L., Qiao L.-Y., Wang W.-Y., He S.-H. (2009). Purification, characterization and biological activities of the l-amino acid oxidase from *Bungarus fasciatus* snake venom. Toxicon.

[65] Markland F.S., Swenson S. (2013). Snake venom metalloproteinases. Toxicon.

[66] Li Xuehong W.S., Jiewei G., Lei W., Canzhong Z. (2024). Analysis on the Treatment of a Severe Child Patient Bitten by *Bungarus suzhenae*. China J. Emerg. Resusc. Disaster Med..

[67] Shah A., Ahmed M., Sher N., Mushtaq N., Khan R.A. (2021). Efficacy of Silene arenosa extract on acetylcholinesterase in *Bungarus sindanus* (*krait*) venom. J. Tradit. Chin. Med..

[68] Zybailov B., Mosley A.L., Sardiu M.E., Coleman M.K., Florens L., Washburn M.P. (2006). Statistical Analysis of Membrane Proteome Expression Changes in Saccharomyces cerevisiae. J. Proteome Res..

[69] Zhou J.-Y., Schepmoes A.A., Zhang X., Moore R.J., Monroe M.E., Lee J.H., Camp D.G., Smith R.D., Qian W.-J. (2010). Improved LC−MS/MS Spectral Counting Statistics by Recovering Low-Scoring Spectra Matched to Confidently Identified Peptide Sequences. J. Proteome Res..

[70] Rozanova S., Barkovits K., Nikolov M., Schmidt C., Urlaub H., Marcus K., Marcus K., Eisenacher M., Sitek B. (2021). Quantitative Mass Spectrometry-Based Proteomics: An Overview. Quantitative Methods in Proteomics.

[71] Weisbrod C.R., Eng J.K., Hoopmann M.R., Baker T., Bruce J.E. (2012). Accurate Peptide Fragment Mass Analysis: Multiplexed Peptide Identification and Quantification. J. Proteome Res..

[72] Kang T.S., Georgieva D., Genov N., Murakami M.T., Sinha M., Kumar R.P., Kaur P., Kumar S., Dey S., Sharma S. (2011). Enzymatic toxins from snake venom: Structural characterization and mechanism of catalysis. FEBS J..

[73] Billiald P., Senji Laxme R.R., Khochare S., de Souza H.F., Ahuja B., Suranse V., Martin G., Whitaker R., Sunagar K. (2019). Beyond the ‘big four’: Venom profiling of the medically important yet neglected Indian snakes reveals disturbing antivenom deficiencies. PLOS Neglected Trop. Dis..

[74] Habermann E., Hardt K.L. (1972). A sensitive and specific plate test for the quantitation of phospholipases. Anal. Biochem..

[75] Tasoulis T., Lee M.S.Y., Ziajko M., Dunstan N., Sumner J., Isbister G.K. (2020). Activity of two key toxin groups in Australian elapid venoms show a strong correlation to phylogeny but not to diet. BMC Evol. Biol..

[76] Kuch U., Senji Laxme R.R., Attarde S., Khochare S., Suranse V., Martin G., Casewell N.R., Whitaker R., Sunagar K. (2021). Biogeographical venom variation in the Indian spectacled cobra (*Naja naja*) underscores the pressing need for pan-India efficacious snakebite therapy. PLOS Neglected Trop. Dis..

[77] Teng C., Ouyang C., Lin S. (1985). Species difference in the fibrinogenolytic effects of α- and β-fibrinogenases from Trimeresurus mucrosquamatus snake venom. Toxicon.

[78] Maisano M., Trapani M.R., Parrino V., Parisi M.G., Cappello T., D’Agata A., Benenati G., Natalotto A., Mauceri A., Cammarata M. (2013). Haemolytic activity and characterization of nematocyst venom fromPelagia noctiluca(*Cnidaria: Scyphozoa*). Ital. J. Zool..

[79] Qiyi H., Xiong G., Li B., Huang D., Zuo T., Deng Q., Liu J., Zhang Y., Chen D., Luo C. (2017). The Molecular Mechanism Study on the Treatment of Snakebite by Traditional Chinese Herb *Cynanchum paniculatum*. SCIENTIA SINICA Vitae.

